# Hyperbilirubinemia at hospitalization predicts nosocomial infection in decompensated cirrhosis: Data from ATTIRE trial

**DOI:** 10.1097/HC9.0000000000000648

**Published:** 2025-03-21

**Authors:** Harriett Fuller, Thais H. Tittanegro, Alexander A. Maini, Louise China, Freya Rhodes, Natalia Becares Salles, Subhankar Mukhopadhyay, Bernadette Moore, Alastair O’Brien

**Affiliations:** 1 School of Food Science and Nutrition, University of Leeds, Leeds, UK; 2 Division of Medicine, UCL Institute for Liver and Digestive Health, London, UK; 3 Peter Gorer Department of Immunobiology, School of Immunology and Microbial Sciences, King’s College London Strand London, UK; 4 Comprehensive Clinical Trials Unit, University College London, UK

**Keywords:** antibiotic prophylaxis, cholesterol esterification, lipidometabolomics, respiratory tract infections, statins

## Abstract

**Background:**

To identify clinical characteristics and serological biomarkers that predicted subsequent nosocomial infection in ATTIRE trial patients.

**Methods:**

We identified 360 patients at hospitalization without infection and not prescribed antibiotics and compared clinical characteristics between those who subsequently developed a nosocomial infection and not. In a 68-patient subcohort, we compared plasma biomarkers of bacterial translocation, infection, and inflammation at hospitalization between those who developed a nosocomial infection and not. In a 56-patient subcohort, we investigated plasma lipidomic profiles in those who did and did not develop nosocomial infection using Lipotype Shotgun platform analysis and multivariate statistical techniques. To further investigate lipid pathways, we compared outcomes in patients taking statins or not at hospitalization.

**Results:**

Serum bilirubin >188 µmol/L at hospitalization predicted subsequent nosocomial infection in univariate and multivariate analyses, with 80% specificity. The most common nosocomial infections were respiratory tract (29%) and those developing infection had significantly greater 28 and 90-day mortality than those not (*p*=9.34E−05 and 0.014). Serological biomarkers of bacterial translocation, infection, and inflammation did not predict subsequent infection. Partial least squares discriminatory analyses identified cholesterol esters (CEs) (CE.18.1.2, CE.18.1.0, and CE.16.0.0) as important predictors of infection but provided only a small improvement in predictive ability over bilirubin alone. RNA-sequencing analyses suggest this is mediated by a downregulation of the cellular cholesterol esterification enzyme sterol *O*-acyltransferase 1. Statin use was not associated with nosocomial infection prevention.

**Conclusions:**

In ATTIRE, elevated serum bilirubin at hospitalization was the only clinical characteristic that predicted subsequent development of nosocomial infection. Considering the rising incidence of antimicrobial resistance, these data could be used to limit antibiotic prophylaxis or aid trial design for investigating use in high-risk patients.

## Graphical abstract

**Figure:**
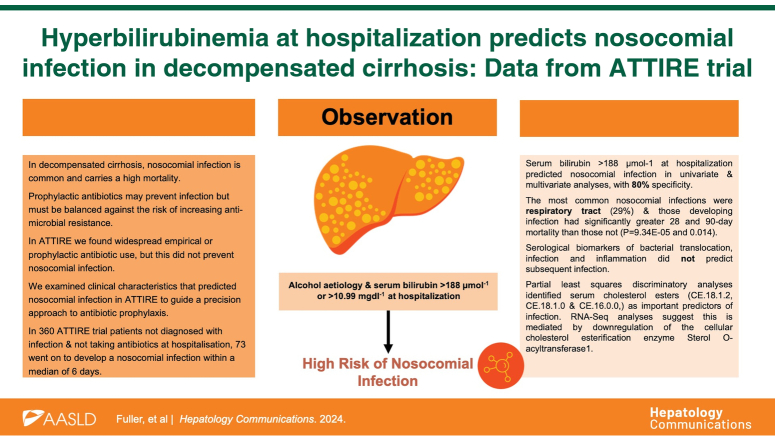

## STUDY HIGHLIGHTS

1. **What is known**:

In patients hospitalized with decompensated cirrhosis, nosocomial infection is common and carries a high mortality. In some clinical scenarios, evidence supports the use of prophylactic antibiotics to prevent infection, but this must be balanced against the risk of increasing antimicrobial resistance. In the ATTIRE trial, we found widespread empirical/prophylactic antibiotic use, but this did not prevent nosocomial infection. This follow-up study examined baseline clinical characteristics that predicted the development of nosocomial infection in ATTIRE patients to guide a more precise approach to antibiotic prophylaxis that might improve outcomes and reduce antimicrobial resistance. As a major potential confounder for nosocomial infection analyses is the widespread use of antibiotics, we selected patients who had not been diagnosed with infection and were not taking antibiotics at hospitalization. We also performed exploratory analyses on subgroups of patients to explore whether serological biomarkers of inflammation, infection, bacterial translocation, and lipid metabolomics on day 1 of hospitalization could predict the subsequent development of nosocomial infection.

2. **What is new here**:

In the 360 ATTIRE trial patients not diagnosed with infection and not taking antibiotics at hospitalization, 73 went on to develop a nosocomial infection within a median of 6 days. The only clinical characteristic at hospitalization that predicted nosocomial infection in univariate and multivariate analyses was elevated serum bilirubin with a threshold >188 µmol/L yielding a specificity of 80% to predict subsequent nosocomial infection. In all, 84/360 patients (23%) had a bilirubin >188 µmol/L at baseline.

Patients who developed nosocomial infection had a 28-day mortality 3 times greater than those who did not (22% compared to 6.3%, *p*=0.00009). Given this poor prognosis, our findings support the use of prophylactic antibiotics in patients hospitalized with decompensated cirrhosis with bilirubin >188 µmol/L and predominant alcohol etiology, which could be used as a key inclusion criterion for future clinical trials aimed at preventing nosocomial infection. However, the most frequently diagnosed infection was lower respiratory tract, which is caused by viruses in up to 25%, and therefore improving vaccination uptake, isolating patients with respiratory infection symptoms, and appropriate airway management in encephalopathic patients to prevent aspiration could be included with antibiotic prophylaxis as a complex intervention to improve outcomes from this dreaded complication.

## INTRODUCTION

Nosocomial infection is a major cause of morbidity and mortality in decompensated cirrhosis.^1^^,^^2^ Clinical guidelines support early antibiotic prescription in suspected infection as mortality risk increases in sepsis if appropriate therapy is delayed^3^^–^^5^; however, evidence regarding antibiotic prophylaxis to prevent infection in hospitalized patients is much less certain. Studies support antibiotic prophylaxis to prevent infection in certain cirrhosis phenotypes, such as alcoholic hepatitis, variceal hemorrhage, and acute-on-chronic liver failure.^6^^–^^8^ However, empirical or prophylactic antibiotic use is far more widespread, as we observed in our ATTIRE trial (Albumin to prevent infection in chronic liver failure, 2016–2019), with half of all antibiotics prescribed at hospitalization given to patients not actually clinically diagnosed with the infection.^9^ Furthermore, this “real-world” antibiotic prescribing approach had no impact on the risk of infection during hospitalization, nor mortality, compared to patients not prescribed antibiotics^10^ and such indiscriminate antibiotic use may worsen antimicrobial resistance,^11^ a global health care challenge. We hypothesized that accurate identification of patients at high risk of nosocomial infections using routinely collected clinical data at hospitalization could enable a more tailored antibiotic prophylaxis approach that might improve outcomes and reduce overprescription.^1^ In addition, combining these data with serological biomarkers, commonly metabolites that represent biological processes within organisms,^12^ may improve prediction accuracy. However, to date, clinical features (eg, fever and tachycardia) and blood tests (eg, white cell count, C-reactive protein, and procalcitonin) aid early diagnosis of infection or sepsis and initiate prompt antibiotic therapy,^13^ but have not been shown to predict those likely to develop an infection during a hospital stay. Low levels of ascitic fluid protein can predict the development of spontaneous bacterial peritonitis, guiding antibiotic prophylaxis in outpatients, but this is not commonly used in hospitalized patients.^14^^,^^15^

So, using ATTIRE trial data and samples, we tested the ability of clinical characteristics, blood tests, markers of bacterial translocation, inflammation, infection, and lipid metabolomics at hospitalization to predict subsequent nosocomial infection. We considered the widespread use of antibiotics and diagnosis of community-acquired infection as major potential confounders to these analyses, and so selected patients who had not been diagnosed with infection and were not taking antibiotics at hospitalization.

## METHODS

### ATTIRE

ATTIRE was a trial of targeted human albumin infusions versus standard care involving 777 hospitalized patients with decompensated cirrhosis^9^ (see Supplemental Figure S1, http://links.lww.com/HC9/B911 and Supplemental Methods, http://links.lww.com/HC9/B911). The result was neutral, and so we pooled all data to study nosocomial infection, which was defined as a new infection that occurred on or after day 3 following trial enrollment. This diagnosis was made by the clinicians attending the patients, which was part of the ATTIRE trial composite primary endpoint, and sites were asked to complete infection case report forms to provide evidence of infection.

### Identification of clinical characteristics to predict the development of nosocomial infection in patients hospitalized with decompensated cirrhosis

**FIGURE 1:**
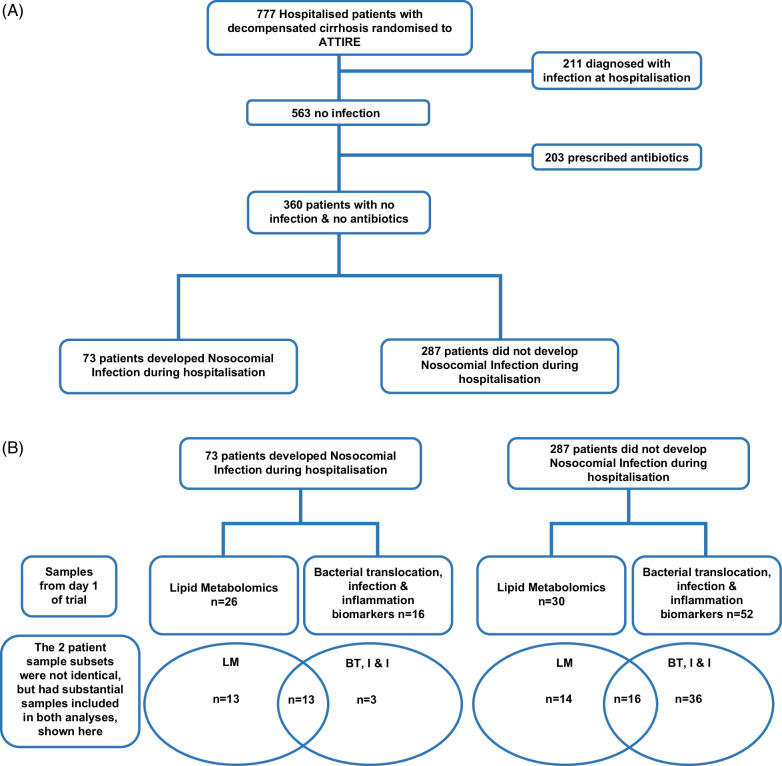
Schema for (A) patients and (B) samples selected for analyses. Abbreviation: ATTIRE, Albumin to prevent infection in chronic liver failure.

We examined baseline characteristics from the 360 ATTIRE patients with no infection and not treated with antibiotics at baseline to determine predictive factors to predict which patients went on to develop nosocomial infection (n=73) during the trial treatment period. See Figure 1A for the schema of patients selected for analyses. Multivariate analyses were also conducted to assess for associations between baseline clinical variables and subsequent nosocomial infection development in these patients. Models included (i) demographic factors and clinical measurements, (ii) model 1 covariates + clinical features of cirrhosis, and (iii) model 2 covariates + medication use (nonselective beta-blockers, proton pump inhibitors, and prednisolone).

### Assessment of biomarkers in plasma taken at randomization (before albumin infusions) to predict the development of nosocomial infection in patients hospitalized with decompensated cirrhosis

#### Markers of bacterial translocation, infection, and inflammation

We measured the following: endotoxin binding protein—LBP; soluble CD14—sCD14; tumor necrosis factor—TNF; interleukins—IL-1, 4, 6, and 8; CD163; CCL8/MCP-2; and procalcitonin—PCT. Data were taken from our published data set of 143 patient samples selected at random by the UCL (University College London) CCTU (Comprehensive Clinical Trials Unit),^16^ of which we identified a subset of 68 ATTIRE patients without infection and not treated with antibiotics at randomization for these analyses, with 16 subsequently developing a nosocomial infection (Figure 1B). Samples were processed using Luminex multiplex assay (R&D Systems) (Supplemental Table S1, http://links.lww.com/HC9/B911). PGE_2_ was measured using the Amersham Prostaglandin E2 Biotrak enzyme immunoassay (EIA) System (GE Healthcare).

#### Lipidometabolomic biomarkers

Plasma was taken at randomization from a subset of 56 patients with no infection and not treated with antibiotics (including rifaximin) at randomization, with an even sex split, of whom 26 developed a nosocomial infection (Figure 1B) but otherwise chosen at random, were analyzed using Lipotype Shotgun untargeted mass spectrometry Lipidomics platform (http://www.lipotype.com/). This covers 13 distinct lipid classes: PC, phosphatidylcholine; PE, phosphatidylethanolamine; PI, phosphatidylinositol; DAG, diacylglycerol; TAG, triacylglycerol; SM, sphingomyelin; CE, cholesteryl ester; LPC, lyso-phosphatidylcholine; LPE, lyso-phosphatidylethanolamine; PC O-, ether-linked PC; PE O-, ether-linked PE; CER, ceramide; and CHOL, cholesterol.

Lipid metabolomics analyses can be limited by the application of traditional univariate approaches that study the targeted metabolite(s) of interest.^17^ We therefore used unsupervised (principal component analysis) and supervised (partial least squares discriminatory analysis [PLSDA]) multivariate statistical methods to investigate metabolites. For PLSDA, a variable of importance in projection (VIP) score is assigned to each variable based upon how much variation in the outcome that variable accounts for. A VIP score ≥1 is a good discriminatory variable (see Supplemental Methods, http://links.lww.com/HC9/B911 for further details).

We performed exploratory analyses to investigate the potential role of lipids in mediating infection in decompensated cirrhosis:

- Effect of use of lipid-lowering drugs on infection: We compared baseline characteristics and incidence of infection, renal dysfunction, and mortality between patients prescribed statins or not at hospitalization (Supplemental Table S2, http://links.lww.com/HC9/B911 and Supplemental Methods, http://links.lww.com/HC9/B911), for further information.
- Gene regulation of lipid metabolic enzymatic pathways: We performed bulk RNA sequencing (RNA-seq) of venous whole blood in non-ATTIRE patients (at the Wellcome Sanger Institute) stimulated with/without 1 ng/mL lipopolysaccharide (LPS) (*Salmonella abortus equi* S-form [TLR *grade*], Enzo Life Sciences), for 4 to simulate an infectious stimulus. Samples were taken from healthy volunteers (HV, n=5), outpatients with refractory ascites (ORA) attending for day-case paracentesis (Royal Free Hospital, n=5), and hospitalized patients with acute decompensation (AD, n=10, University College Hospitals London—UCLH). Four ADs were receiving antibiotics. No ORA patient had an infection at sampling, before albumin and paracentesis, with none taking antibiotics or statins. HVs (non-smoking), aged 18–50, provided samples for analyses.

### Statistical analysis

All authors vouch for the completeness and accuracy of the data. Our sample size was fixed, and we planned exploratory analyses with no alpha spending to inform future studies.

Graph Pad Prism 8 was used for 2-way ANOVA and bivariate statistical tests, including *t* tests for continuous variables using unpaired for normally distributed data, Mann-Whitney with log-rank testing for non-normally distributed, and chi-squared for categorical variables. Multivariate regression analyses, principal component analysis, and PLSDA analyses were conducted in R studio (version 4.2.1).

### Ethics

ATTIRE was approved by the London-Brent Research Ethics Committee (ref15/LO/ 0104) and the Medicines and Healthcare Products Regulatory Agency (ref:20363/0350/001-0001). Written informed consent was obtained from patients. For incapacitated patients, a legal representative provided written informed consent until the patient regained capacity.

For RNA-seq analyses, NHS Research Ethics Committee ethical approval was given for “An investigation of suppression of cirrhosis-mediated immune suppression by prostaglandin receptor antagonism” (IRAS:170839, REC:15/LO/0800) (Alastair O’Brien, principal investigator and UCLH joint research office sponsor). All procedures are in accordance with the Helsinki Declaration of 1975, revised in 1983. This covered patient and healthy volunteer recruitment, with further approval from the Health Research Authority in 2016.

## RESULTS

### Elevated serum bilirubin at hospitalization predicted subsequent nosocomial infection in patients not diagnosed with infection nor prescribed antibiotics

The median time from trial entry to nosocomial infection diagnosis was 6 days. The sites of infection were the respiratory tract (21 patients, 29%), 10 urinary tracts (13.7%), 6 skin/soft tissue, spontaneous bacterial peritonitis, and bacteremia each (6 each, 8%) and 1 fungal, with 23 (31.5%) unknown/not described. A microorganism was reported for 21/73 infections, with 15 sensitive organisms, 2 resistant, 3 resistance status not reported, and 1 influenza A (fungal organism not reported) (Supplemental Table S3, http://links.lww.com/HC9/B911). There were 11 Gram-positive and 9 Gram-negative organisms. Only 1 respiratory infection reported a bacterium, and none were reported in the unknown/not described category. Patients not diagnosed with infection nor prescribed antibiotics who developed nosocomial infection had significantly greater 28-day and 90-day mortality compared to those who did not (*p*=9.34E−05 and 0.014), but not 180 days (Table 1). There were no apparent differences in mortality comparing infection types, with numbers too small to perform robust statistical analyses and no differences in mortality between infections in which a microorganism was reported or not (28-day—*p*=0.8; 90-day—*p*=0.58; 180-day—*p*=0.90; chi-squared test) (Table 2).

**TABLE 1:** Baseline clinical characteristics of the 360 patients without infection and not prescribed antibiotics at the ATTIRE trial baseline

	Developed nosocomial infection (n=73)	Did not develop nosocomial infection (n=287)	*p*
Male, n (%)	50 (68.5)	202 (70.4)	0.75
Alcohol-induced cirrhosis, n (%)	68 (93)	258 (89.9)	0.40
MELD	20.62 (18.35–221)	19.55 (17.95–20.35)	0.08
Bilirubin (μmol/L)	125 (102–187)	103 (91–111)	0.02
Creatinine (mmol/L)	65 (60–69)	63 (60–68)	0.81
INR	1.7 (1.5–1.7)	1.6 (1.6–1.7)	0.41
Albumin (g/L)	23 (22–24)	24 (24–25)	0.08
WCC (×10^9^/L)	6.9 (5.5–8)	6.9 (6.5–7.5)	0.62
CRP (mg/L)	20.5 (14–33)	20 (18–22)	0.70
Age (y)	53.4 (51–55.8)	53 (51.7–54.2)	0.77
Presence of ascites, n (%)	43 (58.9)	192 (66.9)	0.20
Albumin treatment group, n (%)	39 (53)	140 (49)	0.48
PPI use, n (%)	37 (50.7)	154 (53.7)	0.65
NSBB use, n (%)	15 (20.5)	57 (19.9)	0.90
Prednisolone use, n (%)	17 (23.3)	45 (15.7)	0.12
Rifaximin use, n (%)	17 (23.3)	38 (13.2)	0.03
Alcoholic hepatitis, n (%)	25 (34.2)	72 (25.1)	0.12
HE—grade III/IV, n (%)	9 (12.3)	41 (14.3)	0.56
VB, n (%)	5 (6.9)	17 (5.9)	0.85
28-d mortality	16/73 (22%)	18/287 (6.3%)	9.34E−05
90-d mortality	21/73 (29%)	44/287 (15%)	0.014
180-d mortality	27/73 (37%)	72/287 (25%)	0.075

*Note*: Data are shown as mean (95% CI) for age with unpaired *t* test as normally distributed but median (95% CI) for other clinical variables with Mann-Whitney *t* test, as data not normally distributed. Differences between gender, use of PPI/NSBB/prednisolone, alcoholic hepatitis, variceal bleeding, and mortality diagnosis were assessed using chi-squared testing.

Abbreviations: ATTIRE, albumin to prevent infection in chronic liver failure; CRP, C-reactive protein; INR, international normalised ratio; NNBB, nonselective beta-blockers; PPI, proton pump inhibitor; VB, variceal bleed; WCC, white cell count.

**TABLE 2:** 28- to 180-day mortality according to different types of nosocomial infections in ATTIRE trial patients with no infection at baseline and not prescribed antibiotics at hospitalization

Infections	28-d mortality	90-d mortality	180-d mortality
Lower respiratory tract	3/21 (14%)	4/21 (19%)	6/21 (29%)
UTI	2/10 (20%)	3/10 (30%)	4/10 (40%)
SBP	1/6 (16.7%)	1/6 (16.7%)	2/6 (33.3%)
Spontaneous bacteremia	2/6 (33.3%)	2/6 (33.3%)	2/6 (33.3%)
Skin/soft tissue	1/6 (16.7%)	2/6 (33.3%)	2/6 (33.3%)
Unknown/not described	7/23 (30.4%)	9/23 (39.1%)	11/23 (47.8%)
Fungal	0/1	0/1	0/1
All with pathogen isolated	5/21 (23.8%)	7/21 (33.3%)	8/21 (38%)
All without pathogen isolated	11/52 (21.2%)	14/52 (26.9%)	19/52 (36.5%)

Abbreviations: ATTIRE, Albumin to prevent infection in chronic liver failure; SBP, spontaneous bacterial peritonitis; UTI, urinary tract infection.

The only clinical characteristic at hospitalization that significantly differed between patients who went on to develop nosocomial infections or not was serum bilirubin, with a median of 125 μmol/L (95% CI: 102–187) in those who developed infection and 103 μmol/L (95% CI: 91–111) in those who did not, *p*=0.02 (Table 1). Furthermore, in multivariate analyses including baseline clinical variables (including prescribed medications) as covariates, serum bilirubin was the only variable associated with the development of nosocomial infection (*p*=0.013) (Table 3 and Supplemental Table S4, http://links.lww.com/HC9/B911). In post hoc follow-up analyses, Youden’s index was calculated to determine an optimum threshold for bilirubin levels. Using Youden’s index,^18^ we identified that a bilirubin threshold of 188 μmol/L or above in the 360 patients without infection and no antibiotics at baseline predicted subsequent nosocomial infection with a specificity of 80%, OR: 2.18 (95% CI: 1.25–3.82), *p*=0.006 and this applied to 23% (84/360) of our study population with a sensitivity of 36% (see Supplemental Results, http://links.lww.com/HC9/B911 for the specificity of a range of bilirubin values to predict nosocomial infection). Following the replacement of bilirubin levels with this binary threshold of >188 μmol/L in the multivariate model, this threshold was also found to be highly significantly associated with nosocomial infection (*p*=0.004, Supplemental Table S5, http://links.lww.com/HC9/B911).

**TABLE 3:** Multivariate model to investigate the ability of clinical characteristics of the 360 patients without infection and not prescribed antibiotics at ATTIRE trial baseline to predict subsequent nosocomial infection

Variable	β	SE	OR	95% lower	95% upper	*p*
Intercept	−2.153	1.345	0.116	0.008	1.579	0.110
Bilirubin (μmol/L)	0.003	0.001	1.003	1.001	1.005	0.013
Age (y)	0.014	0.016	1.014	0.983	1.046	0.376
Sex	−0.013	0.323	0.987	0.515	1.842	0.968
Albumin (g/L)	−0.023	0.041	0.978	0.903	1.061	0.581
Creatinine (mmol/L)	−0.001	0.004	0.999	0.991	1.006	0.705
WCC (×10^9^/L)	0.028	0.034	1.028	0.959	1.096	0.409
CRP (mg/L)	0.000	0.007	1.000	0.985	1.015	0.969
VB	−0.042	0.614	0.959	0.252	2.956	0.945
Presence of ascites	−0.266	0.314	0.766	0.416	1.430	0.397
HE	−0.315	0.467	0.730	0.272	1.740	0.500
NSBB use	0.094	0.391	1.099	0.493	2.313	0.810
PPI use	0.202	0.305	1.223	0.677	2.245	0.508
Prednisolone use	0.129	0.400	1.137	0.508	2.456	0.748

Abbreviations: ATTIRE, Albumin to prevent infection in chronic liver failure; CRP, C-reactive protein; NNBB, nonselective beta-blockers; PPI, proton pump inhibitor; VB, variceal bleed; WCC, white cell count.

We found no impact of the use of proton pump inhibitors, nonselective beta-blockers, or prednisolone at hospitalization on nosocomial infection risk in these patients. There was a significantly higher incidence of nosocomial infection in patients prescribed rifaximin at hospitalization (*p*=0.03). Of note, albumin treatment had no effect on the development of nosocomial infection (Table 1).

### Serological biomarkers of bacterial translocation, infection, and inflammation at hospitalization did not predict the development of nosocomial infection

In a subcohort of 68 patients (see Supplemental Table S6, http://links.lww.com/HC9/B911 for clinical characteristics), no differences in IL1-β, IL-6, IL-10, TNF-α, IL-4, IL-8, PCT, LBP, sCD14, CD163, CCL8/MCP-2, and PGE_2_ at hospitalization were observed in patients who subsequently developed infection (n=16) or not (n=52, Table 4).

**TABLE 4:** Plasma markers of bacterial translocation, inflammation, and infection in a 68-patient subcohort of those with no infection at baseline and not treated with antibiotics with samples taken at baseline

Biomarker	Developed nosocomial infection (N=16)	Did not develop nosocomial infection (N=52)	*p*
IL1-β (pg/mL)	0 (0–1.3)	0 (0–0.2)	0.51
IL-6 (pg/mL)	13.05 (5.9–24.6)	11.9 (8.3–14.9)	0.51
IL-10 (pg/mL)	0 (0–0)	0 (0–0)	0.20
TNF-α (pg/mL)	3.45 (2.4–6.1)	3.7 (3.4–4.7)	0.59
IL-4 (pg/mL)	0 (0–0)	0 (0–0)	0.41
IL-8 (pg/mL)	53.9 (13.3–253.5)	115 (95.5–167)	0.66
PCT (ng/mL)	121.3 (32.1–214.5)	128.7 (95.5–167)	0.52
LBP (ng/mL)	1765 (1020–4590)	1695 (1330–2370)	0.93
sCD14 (ng/mL)	1865 (934.4–8790)	2860 (1670–5380)	0.57
CD163 (ng/mL)	2802 (1758–3691)	2591 (2237–3282)	0.91
CCL8/MCP-2 (pg/mL)	44.1 (17.9–63.1)	50.5 (46.1–54.8)	0.50
PGE_2_ (pg/mL)	663.8 (386–1391)	711.6 (558.9–1002)	0.86

*Note*: Data presented as medians and 95% CIs with Mann-Whitney *t* test.

Abbreviations: LBP, lipopolysaccharide-binding protein; PCT, procalcitonin.

### Plasma lipid metabolomic profiling at hospitalization predicted subsequent nosocomial infection in patients not diagnosed with infection nor prescribed antibiotics

Clinical characteristics of the lipid metabolomic subcohort (56/360, Supplemental Table S7, http://links.lww.com/HC9/B911) were similar to overall patients, except for sex (70% male overall, 55% male in subcohort), which was intentional due to differences in lipid profiles between sexes.^19^ Overall analyses revealed no significant differences between metabolites when comparing patients who went on to develop nosocomial infection with those who did not, using Student *t* tests (Supplemental Figure S2, http://links.lww.com/HC9/B911).

The principal component analysis highlighted lipids from “CE” (cholesterol esters) and “Chol” (cholesterol) classes to be the main drivers of separation between individuals who did or did not develop nosocomial infection (Supplemental Figure S3, http://links.lww.com/HC9/B911). Baseline PLSDA models, including clinical covariates, explained 19% of the variation between those who developed nosocomial infection or not, with bilirubin being identified as the only important variable (VIP=2.44). The addition of the CE lipid class to PLSDA models containing clinical variables explained an additional 4% of the variation between individuals who did or did not develop nosocomial infection (Table 5). The addition of cholesterol to the model explained an additional 6% of the variation in infection status, but this model was not significant in cross-validation. Across all models, bilirubin was the most important variable in the characterization of infection status, followed by CE.18.2.0; with CE.16.0.0 and CE.18.1.0 also (VIP≥1).

**TABLE 5:** PLSDA results for clinical characteristics and lipid metabolomic profiling from a 56-patient subcohort in Table 2

Model output	Model 1	Model 2	Model 3	Model 4
Outcome variance explained (*R* ^2^ *Y*)	0.19	0.20	0.24	0.31
Outcome variance explained cross-validation (*Q* ^2^ *Y*)	0.13	0.13	0.06	*NA*
*p* value—*R^2^Y*	**0.05**	**0.05**	**0.05**	**0.05**
*p* value—*Q^2^Y*	**0.05**	**0.05**	**0.05**	**0.70**
Clinical variables at hospitalization	**VIPs**			
Bilirubin	**2.44**	**2.52**	**2.37**	**2.68**
Baseline creatinine	0.72	0.85	**1.44**	**1.06**
MELD score	0.47	0.48	**1.09**	0.43
CRP	0.41	0.55	**1.10**	0.47
WCC	0.32	0.53	0.93	0.47
Albumin	0.20	0.37	0.51	0.12
Statins use	0.17	0.24	0.32	0.04
Age	—	0.88	**1.42**	**1.06**
Sex	—	0.42	0.40	0.15
Lipids	**VIPs**			
CE.14.0.0	—	—	0.84	0.28
CE.15.0.0	—	—	0.52	0.35
CE.16.0.0	—	—	**1.50**	**1.05**
CE.16.1.0	—	—	0.29	0.27
CE.17.0.0	—	—	0.53	0.35
CE.17.1.0	—	—	0.36	0.10
CE.18.0.0	—	—	0.65	0.53
CE.18.1.0	—	—	**1.54**	**1.71**
CE.18.2.0	—	—	**2.37**	**2.70**
CE.18.3.0	—	—	0.23	0.12
CE.19.1.0	—	—	0.80	0.23
CE.20.1.0	—	—	0.53	0.43
CE.20.2.0	—	—	0.72	0.54
CE.20.3.0	—	—	0.12	0.07
CE.20.4.0	—	—	0.15	0.13
CE.20.5.0			0.49	0.33
CE.22.1.0	—	—	0.36	0.23
CE.22.6.0	—	—	0.18	0.16
CE.24.1.0	—	—	0.40	0.10
Chol	—	—	—	**2.48**

*Note*: This table highlights key model characteristics and VIP scores across PLSDA models. Significant *p* values and variables designated as important (VIP ≥1) are shown in bold.

Abbreviations: CRP, C-reactive protein; PLSDA, partial least squares discriminatory analysis; VIP, variable of importance in projection; WCC, white cell count.

Area under the curves were created for the following: model 1: baseline bilirubin + creatinine; model 2: model 1 + CE.16.0.0 + CE.18.1.0 + CE.18.2.0; model 3: model 2 + cholesterol. Receiver-operated curves highlighted the prediction of nosocomial infection status in all models in both training and test data sets, with the inclusion of lipids resulting in a small improvement of predictive ability in the models with AUC rising from 0.66 to 0.72 (Supplemental Figure S4, http://links.lww.com/HC9/B911).

In post hoc analyses, we found a strong positive correlation between bilirubin and MELD score^20^; CE.18.2.0, CE.18.1.0, and CE.16.0.0 were inversely correlated with MELD, and cholesterol levels positively correlated (Supplemental Figure S5, http://links.lww.com/HC9/B911).

### Exploratory analyses

#### Use of statins at hospitalization

There were 47 patients—taking simvastatin (23), atorvastatin (21), pravastatin (2), and rosuvastatin (1), with no differences in nosocomial infection according to use. Patients not on statins were younger, had higher bilirubin and MELD scores, but numbers were too small to perform propensity score matching to account for differences (Table 6).

**TABLE 6:** Baseline characteristics and clinical outcomes of patients taking statins at baseline compared with patients not prescribed statins during hospitalization

	Statins (N=47)	% or 95% CI	No statins (N=730)	% or 95% CI	*p*
Age (y)	59.9	56.5–62.3	53.4	52–53.9	<0.0001*
Male	35	74.5%	515	70.5%	0.57
Suspected variceal bleed	6	12.8%	109	14.9%	0.69
Ascites	33	70.2%	488	66.8%	0.63
HE	11	23.4%	138	18.9%	0.45
Infection at randomization	9	19.1%	202	27.7%	0.20
Use of antibiotics	20	42.6%	391	53.6%	0.14
MELD score	16.5	15.2–19.5	19.7	19–20.3	0.01*
Serum albumin (g/L)	24	23–25	24	23–24	0.21
Creatinine (mmol/L)	73	65–86	67	65–69	0.06
WCC (×10^9^/L) (median)	6.9	6.0–8.3	7.5	7.2–7.9	0.42
CRP (mg/L) (median)	28	15–37	24.0	22–28	0.99
Bilirubin (μmol/L)	56	44–99	100	90–106	0.002*
Clinical outcomes					
Incidence of death during hospitalization	4	8.5%	59	8.1%	0.92
Incidence of new infection	9	19.1%	141	19.3%	0.98
Incidence of kidney dysfunction	8	17.0%	89	12.2%	0.33
28-d mortality	5	10.6%	110	15.1%	0.41
90-d mortality	11	23.4%	174	23.8%	0.95
180-d mortality	14	29.8%	237	32.5%	0.70

*Note*: Data are shown as mean (95% CI) for age with unpaired *t* test as normally distributed but median (95% CI) for other clinical variables with Mann-Whitney *t* test, as data not normally distributed. Differences between clinical outcomes were assessed using chi-squared testing.

* *p* values <0.05.

Abbreviations: CRP, C-reactive protein; WCC, white cell count.

#### Bulk RNA-seq lipid pathway gene expression in whole blood before and after LPS

**FIGURE 2:**
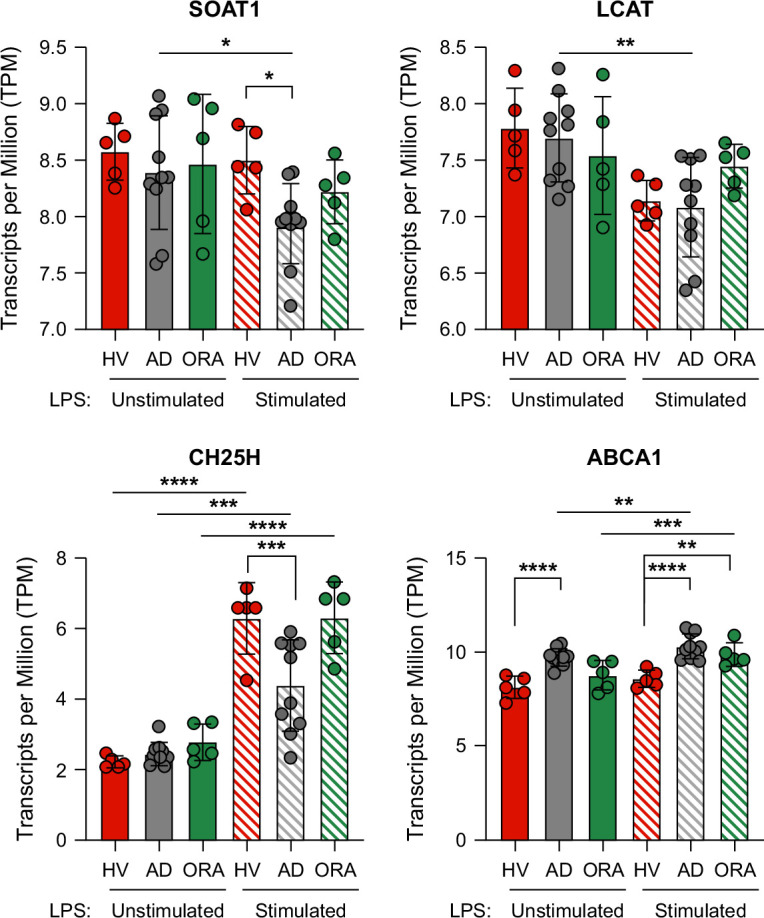
RNA-seq analyses of whole blood untreated and LPS-treated from healthy volunteers (HV, n=5), outpatients with refractory ascites (ORA, n=5), and hospitalized patients with acute decompensation (AD, n=10) for genes involved in lipid metabolomic pathway regulation. Two-way ANOVA with Šídák’s multiple comparisons tests, *p<0.05, **p<0.01, ***p<0.001, ****p<0.0001. Abbreviations: LPS, lipopolysaccharide; RNA-seq, RNA sequencing.

Supplemental Table S8, http://links.lww.com/HC9/B911, describes the clinical characteristics of these non-ATTIRE patients with 85% having an alcohol etiology for liver disease. The cellular cholesterol esterification enzyme sterol *O*-acyltransferase (acyl-Coenzyme A: cholesterol acyltransferase) 1-SOAT1 expression was reduced following LPS stimulation in hospitalized ADs (mean±SD transcripts per million [TPM]: 7.938±0.356) compared to HVs (8.50±0.30, *p*=0.0341) with intermediate expression in ORA (8.221±0.284, Figure 2A). Lecithin cholesterol acyltransferase expression (plasma cholesterol esterification enzyme) was similarly reduced following LPS stimulation in all groups (Figure 2B). The hydroxylation enzyme, cholesterol 25-hydroxylase, was upregulated to a lesser extent in ADs compared to HV following LPS stimulation (*p*<0.001) (Figure 2C). There was an increase in ABCA1, ATP-binding cassette transporter ABCA1 (regulates cellular cholesterol and phospholipid homeostasis), following LPS stimulation in ADs (*p*<0.0001) and ORA (*p*<0.01) compared to HV (Figure 2D). There were no differences in other genes responsible for cholesterol synthesis, hydroxylation, sulfation, or LXR activity before and after LPS stimulation (Supplemental Figure S6, http://links.lww.com/HC9/B911).

PDCD1 (programmed cell death 1) expression was downregulated in ADs compared to HV in LPS-treated whole blood (*p*<0.0001) with no differences in PDCD1LG2 (programmed cell death 1 ligand 2) (Supplemental Figure S7, http://links.lww.com/HC9/B911). In unstimulated blood, IL-10 expression was elevated in ADs compared to HVs (*p*<0.001) with no differences in IL1B, IL-6, and TNF gene expression before and after LPS stimulation (Supplemental Figure S7, http://links.lww.com/HC9/B911).

## DISCUSSION

We demonstrate that in hospitalized patients with decompensated cirrhosis not diagnosed with infection nor prescribed antibiotics, elevated serum bilirubin at hospitalization predicted nosocomial infection during hospitalization in both univariate and multivariate models and selecting those ≥188 μmol/L yielded a specificity of 80%. This threshold accounted for 23% of 360 patients overall. The sensitivity was low, but our aim was to “rule in” subsequent nosocomial infections in a pre-selected population at high risk (ie, “high pre-test probability”). Plasma markers of bacterial translocation, inflammation or infection at hospitalization had no utility to predict nosocomial infection, in line with recent data in a similar cohort.^21^ However, downregulation of blood cholesterol esterification, specifically CE 18.1.0, CE 18.2.0, and CE 16.0.0 did predict nosocomial infection, although the addition of lipid metabolomic data only resulted in a very small improvement of predictive ability over bilirubin alone. Use of proton pump inhibitors, nonselective beta-blockers, steroids, or statins at baseline had no impact on subsequent nosocomial infection, and use of rifaximin was associated with a greater risk of developing nosocomial infection.

The major strengths of our analyses were (1) this was a large cohort from a UK multicenter clinical trial with nosocomial infection diagnosis as part of the composite primary endpoint and (2) our selection criteria avoided the potential confounders of community-acquired infection and/or antibiotic prescription at hospitalization. This approach avoided having to consider differences between clinicians’ antibiotic prescribing habits across the 35 sites. Consistent with other studies, 20% developed nosocomial infection and subsequent prognosis was poor with 28-day mortality 3 times higher and 90-day mortality twice as high as those who did not develop infection.^22^ Combined with our previous study, in which empirical/prophylactic antibiotics at hospitalization did not prevent overall nosocomial infection in decompensated cirrhosis,^10^ these data support limiting the use of prophylactic antibiotics to those with bilirubin ≥188 µmol/L, which could reduce overall antibiotic prescribing and could be a key inclusion criterion for a pragmatic clinical trial to prevent nosocomial infection.

The association between increased incidence of nosocomial infection and rifaximin use was perhaps surprising; certainly, our results support no evidence of benefit to prevent nosocomial infection. Rifaximin is licensed for use in the treatment of chronic low-grade HE (impairing quality of life) and/or the secondary prevention of recurrent overt HE in patients with cirrhosis who have failed standard therapy. Therefore, the patients taking rifaximin were likely to have been previously hospitalized for complications of cirrhosis which would increase their subsequent risk of nosocomial infection during a further hospital admission.^23^ The recent LiverHope study showed that use of rifaximin and simvastatin in decompensated cirrhosis was not associated with reduced development of acute-on-chronic liver failure, liver related complications, or death.^24^ Rifaximin is an effective treatment for HE,^25^ but its efficacy to prevent infection has not been shown in an adequately powered clinical trial, and our data do not support a protective effect for rifaximin regarding nosocomial infection.

There was an even split between Gram-positive and Gram-negative bacteria, and therefore, broad-spectrum antibiotic prophylaxis might prevent urinary tract infection, spontaneous bacterial peritonitis, and bacteremia. However, the most frequently diagnosed infection was the lower respiratory tract, which had an equally poor outcome as other infections, with culture information very limited, consistent with known difficulties collecting pathogens from sputum.^26^ Indeed, studies have shown that up to 25% of nosocomial respiratory infections are viral,^27^^,^^28^ and therefore improving vaccination uptake, isolating patients with respiratory infection symptoms, and appropriate airway management in encephalopathic patients to prevent aspiration may have a significant impact on the prevention of nosocomial infection. Indeed, a more “complex intervention” that incorporates all these approaches may have increased efficacy over antibiotics alone.

Downregulation of blood cholesterol esterification, specifically CE.18.1.0, CE.18.2.0, and CE.16.0.0 predicted nosocomial infection. These data may reflect an increased risk of infection associated with increasing liver disease severity with plasma cholesterol esterification fraction previously shown to be superior to the MELD components to predict 3-month survival in cirrhosis.^29^ The authors ascribed this to a reduction in the activity of the plasma cholesterol esterification enzyme lecithin cholesterol acyltransferase,^30^ which is produced by the liver. The close inverse correlation of CE.18.1.0, CE.18.2.0, and CE.16.0.0 with MELD is consistent with this hypothesis, although we found no differences in whole-blood lecithin cholesterol acyltransferase gene expression between healthy volunteers and patients with cirrhosis. We did observe downregulation of the cellular cholesterol esterification enzyme, SOAT1 in LPS-stimulated whole blood from patients hospitalized with decompensated cirrhosis compared to healthy volunteers and with patients with refractory ascites having intermediate expression. Interestingly, inhibiting SOAT1 in a RAW 264.7 macrophage cell line significantly reduced the upregulation of multiple inflammatory genes following LPS stimulation,^31^ and a myeloid-specific SOAT1 knockout mouse model showed adipose tissue macrophages were polarized toward anti-inflammatory, M2-like phenotypes.^31^ Immune dysregulation is widely reported in patients with advanced liver disease, and our gene expression data showed significant differences in IL-10 and PDCD1 gene expression between patients with AD and healthy volunteers. These data raise the possibility that downregulation of SOAT1 may have a functionally significant role in nosocomial infection susceptibility in cirrhosis. However, it must be acknowledged that these data should be regarded as exploratory at present.

The addition of cholesterol to predictive models of nosocomial infection was not significant in cross-validation and statin use had no effect on nosocomial infection incidence, suggesting these drugs do not prevent infection. Although relatively small cohorts, these data are consistent with a retrospective study demonstrating infection was not reduced in patients with cirrhosis taking statins,^32^ and LiverHope showed simvastatin plus rifaximin did not reduce progression to acute-on-chronic liver failure or mortality in decompensated cirrhosis.^24^ Given their established adverse event profile, we suggest statins are best avoided in decompensated cirrhosis.

The major limitation of our study is a lack of a validation cohort, but we look forward to examining hyperbilirubinemia in our ASEPTIC (primary antibiotic prophylaxis using co-trimoxazole to prevent SpontanEous bacterial PeritoniTIs in Cirrhosis) randomized controlled trial cohort of 442 patients with cirrhosis and ascites, once the primary analyses have been published in 2025.^33^ A further limitation is that our cohort was almost all alcohol-associated, who typically have greater serum bilirubin, and findings may not be relevant to other causes of liver disease. The diagnosis of alcoholic hepatitis was made clinically by site teams, and we have no data on liver biopsy. Our microbial data should also be interpreted with caution as this was not mandated in ATTIRE and so cannot be regarded as comprehensive. Most patients were ward-based and these findings may not apply to those in intensive care. We did not have data on the use of urinary catheters or mechanical ventilation, which are potential confounders although only 2.3% of the overall cohort was admitted to intensive care at baseline. For the subcohort in which we investigated serological markers of bacterial translocation, inflammation, or infection at hospitalization, there were no significant differences in any clinical variables, including bilirubin, between those who developed an infection or not and this cohort may not have been truly representative of the overall patients. The numbers in training and testing models for lipid metabolomics were very small and had twice the incidence of nosocomial infection compared to the whole study population, which might overestimate the AUROC, and these results should be regarded as exploratory. The RNA-seq cohort was not from ATTIRE, although similarly high numbers of alcohol-induced cirrhosis, and these should also be regarded as exploratory.

In summary, we have demonstrated that in ATTIRE trial patients at hospitalization not diagnosed with infection nor treated with antibiotics, serum bilirubin of ≥188 μmol/L predicted nosocomial infection. The biomarkers investigated did not substantially improve predictive ability when compared to bilirubin alone. We suggest that, given the rising incidence of antimicrobial resistance,^23^^,^^34^ these data could be used to limit antibiotic prophylaxis in hospitalized patients or aid trial design for investigating use in those at high risk. However, the high proportion of respiratory tract infections observed supports the notion that prophylaxis needs to be augmented by nonantibiotic measures to have the greatest impact.

## Supplementary Material
