# Supplementary material for: Hyperbilirubinemia at hospitalization predicts nosocomial infection in decompensated cirrhosis: Data from ATTIRE trial
Source: Hepatol Commun. 2025 Mar 21;9(4):e0648. doi: 10.1097/HC9.0000000000000648 (PMC11927652; doi:10.1097/HC9.0000000000000648)
Supplement: Supplementary file 1 [file hc9-9-e0648-s001.docx]

**ATTIRE Trial Investigators**

**Independent Data Monitoring Group:** Professor Dominique Valla (CHAIR), Tim Clayton and Professor Vipul Jairath.

**Data centre at University College Comprehensive Clinical Trials Unit (UCL CCTU):** Kate Bennett, Scott Bevan, James Blackstone, Kashfia Chowdhury, Zainib Shabir and Simon Skene.

**Trial Steering Committee:** Professor Stephen J. Brett (CHAIR), John Crookenden (Patient Representative), Professor Shahid A. Khan, Brennan Kahan, Professor Graeme Alexander, Professor Humphrey Hodgson and Professor Mike Murphy.

**Trial Management Group:** Dr Louise China, Dr Ewan H Forrest, Dr Yiannis Kallis, Jim Portal, Professor Stephen Ryder and Dr Gavin Wright.

**UCL CCTU:** Dr Ana Arbeloa del Moral, James Blackstone, Kashfia Chowdhury, Dr Ana Carolina Estevao, Rosie Hamilton, Ms Khadra Mohamoud and Dr Nicola Muirhead.

**Research Steering Committee:** Professor Mauro Bernardi (CHAIR), Paula Milton (Department of Health and Social Care representative) and Nicola Shepherd (Wellcome Trust representative).

**Microbiology and Adverse Event Review Panel:** Dr Indran Balakrishnan, Dr Mark McPhail, Dr Brian Hogan and Dr Jane Abbott.

**ATTIRE Site Investigators**

Professor Aftab Ala, Dr Richard Aspinall, Dr Andrew Austin, Dr C Lye Ch'ng, Dr Jeremy Cobbold, Dr Lynsey Corless, Dr Alexandra Daley, Professor Matthew Cramp, Dr Ahmed Elsharkawy, Dr Alex Evans, Dr Shaun Greer, Dr Mathis Heydtmann, Dr Coral Hollywood, Dr Peter Isaacs, Professor Rajiv Jalan, Dr Yiannis Kallis, Dr Richard Keld, Dr Andrew King, Dr Stuart McPherson, Dr Judith Morris, Professor Jane Metcalf, Dr Richard Parker, Dr Janisha Patel, Dr Francisco Porraz-Perez, Dr Praveen Rajasekhar, Dr John Ramage, Dr Paul Richardson, Dr Dariush Sadigh, Dr Deepak Suri, Dr Esther Unit, Professor Sumita Verma and Dr Earl Williams.

**ATTIRE Clinical Trial Sites**

Basildon, Basingstoke, Berkshire, Birmingham, Blackpool, Bournemouth, Bristol, Brighton, Coventry, Derby, Durham, Glasgow RI, Glasgow QE, Glasgow RA, Gloucestershire, Heartlands, Hull, Leeds, Liverpool, Manchester, Newcastle, North Tees, North Tyneside, Nottingham, Oxford, Plymouth, Portsmouth, Royal Free, Royal London, South Tyneside, Southampton, Surrey, Swansea, Whittington and Wigan.

**Role of the funder**

The ATTIRE trial and sample collection was funded by the Health Innovation Challenge fund awarded to Dr O’Brien (Wellcome Trust and Department of Health and Social Care) HICF reference HICF-R8-439, WT grant number WT102568.

The lipid metabolomic work was funded by the NIHR UCL Hospitals Biomedical Research Centre (BRC), project grant: “Repurposing of Simvastatin As An Immune-Restorative Therapy In Advanced Liver Disease”.

AOB receives a proportion of his salary from the UCLH NIHR BRC.

Funding sources had no role in the design of this study and will not have any role during its execution, analyses, interpretation of the data, or decision to submit results.

**Contributors:** *Databases were created by HF, LC, AM and TT and verified by AOB. HF, TT, AM, BM and AOB performed statistical analyses. AOB wrote manuscript first draft, with contributions from all authors. All authors read and approved the final version of the manuscript.*

Data sharing can be made available upon reasonable request.

Potential competing interests: There are no conflicts of interest.

**Supplementary Methods**

***ATTIRE Trial.***

ATTIRE was a multicentre, randomized, open-labelled trial to evaluate the effect of daily intravenous 20% human albumin infusions to raise and maintain serum albumin >30 g/L compared to standard medical care in treatment of decompensated cirrhosis patients hospitalized with acute complications and albumin <30 g/L. Patients were aged >18 years, hospitalized with a clinical diagnosis of acute complications of decompensated cirrhosis and serum albumin <30 g/L within 72 hours after hospital admission (as early therapy was more likely to be beneficial) and anticipated hospital length of stay >5 days at randomization. Patients hospitalized with community-onset infection were eligible as they have high rates of nosocomial infection. Recruitment was between 25-Jan-2016 to 28-Jun-2019, at 35 hospitals across England, Scotland and Wales. Key exclusion criteria were advanced hepatocellular carcinoma with life expectancy <8 weeks and patients receiving palliative care.

***Plasma samples.***

Samples were taken using 9mL lithium heparin tubes that were labelled with the patient’s trial ID and day of sample collection and transferred to the site’s hospital laboratories and spun at 1300x g at 20 °C. The plasma layer was removed and frozen at -80 °C in 2mL cryovials with the corresponding trial identifier. Samples were collected from patients at 33 UK hospital sites. They were transferred to University College London at the end of the recruitment period in 2019. All analyses were conducted after first sample thaw.

We used a bead based multiplex assay that allowed accurate, concurrent measurement of multiple analytes in a small volume of sample. Briefly, after defrosting, samples were centrifuged at 16,000 g for 6 minutes and then diluted in calibrator diluent RD6-52 (1:2 for all analytes apart from sCD14 and LBP in which assays plasma was diluted to 1:200). Standards were made up as per the specific product sheet and diluted 1:3 serially to produce a standard curve with the range of detection. Samples and standards were plated using the supplied opaque plate and the microparticle cocktail was added as per instruction. The plate was then sealed with foil and left overnight (14-16 hours) at 4°C on an orbital shaker at 900 rpm. Plates were washed with the addition of a plate magnet and antibody cocktail for the same analytes was added with the plate left on the orbital shaker at 900 rpm for 1 hour at room temperature. Plates were washed again, with the use of a magnetic plate, and streptavidin-phycoerythrin conjugate was added and the plates placed on the orbital shaker at 900 rpm for 30 minutes. The plate then underwent a final wash procedure, and the remaining particles were then resuspended in wash buffer, placed on the orbital shaker at 900 rpm for 5 mins, and read on a Bio-rad Bio-plex reader to determine individual cytokine concentrations interpolated from a standard curve of known concentrations.

***Identification of potential lipid biomarkers to predict subsequent development of nosocomial infection in patients hospitalized with decompensated cirrhosis analysed via unsupervised (principal component analysis (PCA)) and supervised (partial least squares discriminatory analysis (PLSDA) multivariate statistical methods.***

Lipids missing ≥25% of data were excluded resulting in 249 lipids for analysis. Missing data for the remaining lipids were imputed with optimized multiple imputation via iterative PCa (100 simulations, K-fold cross validation) via the missMDA package in R^2^. Normality of lipid distributions was assessed via a Shapiro Wilks test. When non-normally distributed (n=232), lipids were log-normalized. Shapiro Wilks tests were implemented again to assess for normality. When a log transformation did not sufficiently normalise the data (n=51), a square root transformation was implemented and finally a normal score transformation via the blom function in R. If data still appeared non-normal after a square root transformation. Data were pareto scaled and mean centred prior to PCA analysis. PCA models were initially performed on the full lipid panel and then in each lipid subclass individually to investigate if any lipid class contributed to the separation of individuals who developed infection from those that did not to reduce the lipid panel in subsequent analyses due to the small sample size.

Following PCA, lipids identified to contribute to the separation of individuals based on subsequent nosocomial infection status were added to PLSDA models alongside clinical covariates. The supervised dimensionality reduction technique partial least squares discriminatory analysis (PLSDA) determines if any variables contribute to the separation of the defined outcome groups. For this, a variable of importance in projection (VIP) score is assigned to each variable based upon how much variation in the outcome that variable accounts for. A VIP score ≥1 is widely seen as a good discriminatory variable. PLSDA models were considered to significantly differentiate between outcome groups (i.e., infected vs non infected) when the value for both R^2^ (pR^2^) and Q^2^ were ≤0.05 (7-fold cross validation). When >1 component was significantly predictive the number of components to include in the PLSDA model was selected based upon the minimisation of pR2, the minimisation of Q^2^, the minimisation of root mean squared error of estimation (RMSEE) and the maximisation of R^2^. Model 1 included the MELD score, WCC, CRP, albumin, creatinine, bilirubin, and statin use at baseline. Model 2 included model 1 variables alongside demographic variables (age + sex). Lipid classes determined to be important in PCA models were then sequentially added to PLSDA models to determine the improvement on prediction.

Finally, the predictive ability of identified lipids was also assessed through logistic regression models and the generation of a receiver operator characteristic curve, with predictive performance assessed by area under the curve (AUC) values. The training dataset included 60% of cases/controls (n=16 infections, 18 non-infections) and testing dataset included the remaining 40% (n=10 infections, 12 non-infections). To reduce overfitting, only variables identified in PLSDA as ‘important’ were included in each model. An additional model was constructed based on important clinical characteristics, with additional models including important lipids in each identified lipid class. Analyses involving lipidomics data were performed in R studio version 4.21.

***Extraction of data on patients taking Statins at hospitalisation*.**

Statin use was extracted from the concomitant medication (ConMed) case report forms (CRFs). Data included name, dose, start/stop date for all medications during trial from drug charts and were inputted into the ATTIRE database at UCL Comprehensive Clinical Trials Unit. From the list created we filtered the following terms for Statins: 'simvastin','atorvastin','atorvastatin','simvastatin', 'simuastatin','pravastatin', 'rosuvastatin'. A data set was created containing all patients taking the medication matching the terms above. For medication at baseline, we considered entries that had a start date before the randomization date or up to 1 day after. We excluded entries with 2 days or more after randomization date. We only considered entries with a start date and only considered first randomisations. Infection was defined according to the attending clinician’s diagnosis, and, for nosocomial infections, sites were asked to complete infection CRFs with supporting clinical, biochemical, microbiological and radiological data.

Renal dysfunction was defined as serum creatinine increase ≥50% at the time of sample collection compared to randomisation, a rise in serum creatinine ≥0.3 mg/dL within 48 hours or patient initiated on renal replacement therapy (patients receiving renal replacement at baseline could not reach this outcome). HE was defined as grade III or grade IV encephalopathy using the Westhaven Criteria (based on modified components of the Chronic LIver Failure-Sequential Organ Failure Assessment (CLIFSOFA) score, (**Supplementary Table 2)**.

**Supplementary Results: Exploring different serum bilirubin thresholds at hospitalisation to predict nosocomial infection.**

Using SPSS (v29, 2023), Youden’s index identified a bilirubin threshold of 179.5, producing a specificity of 78% and this would apply to 91/360 (25.3%) of the study population who had bilirubin at or above this threshold. Applying a slightly higher bilirubin threshold of 188 would increase the specificity to 80%, without compromising the sample size (it would still be applicable to 23% of the study population (84/360).

**1.** Youden’s bilirubin threshold = 179.50, which gives sensitivity of 0.397, and specificity of 0.784. This applies to 91/360 = 25% (25.3%) of ATTIRE trial population at baseline that were not diagnosed with infection, nor treated with antibiotics.

| Positive if bilirubin greater/equal to at hospitalisation | Sensitivity | 1 - Specificity | Youden's Index |
| --- | --- | --- | --- |
| 179.50 | .397 | .216 | .181 |

**2.** 80% Specificity applies to 84/360 = 23% (23.3%).

| Positive if bilirubin greater/equal to at hospitalisation | Sensitivity | 1 - Specificity | Youden's Index |
| --- | --- | --- | --- |
| 188.00 | .356 | .202 | .154 |

Logistic regression using threshold bilirubin of 188:


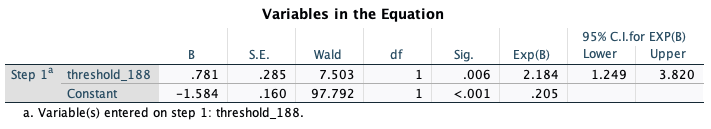


**3.** 85% Specificity.

| Positive if bilirubin greater/equal to at hospitalisation | Sensitivity | 1 - Specificity | Youden's Index |
| --- | --- | --- | --- |
| 222.00 | .274 | .153 | .121 |

**4.** 90% Specificity.

| Positive if bilirubin greater/equal to at hospitalisation | Sensitivity | 1 - Specificity | Youden's Index |
| --- | --- | --- | --- |
| 290.50 | .219 | .105 | .115 |

**Supplementary Figure 1.** ATTIRE patient recruitment and treatment protocol.

(Taken from China L, Skene SS, Bennett K, et al. ATTIRE: Albumin To prevenT Infection in chronic liveR failurE: study protocol for an interventional randomised controlled trial. BMJ Open 2018;8:e023754. doi:10.1136/ bmjopen-2018-023754)

**Supplementary Figure 2.** Overall lipid metabolomic analyses for patients that went on to develop nosocomial infections compared with those that did not. There were no significant differences in lipid classes at baseline between patients that went on to develop infection or not using Student's t-test.


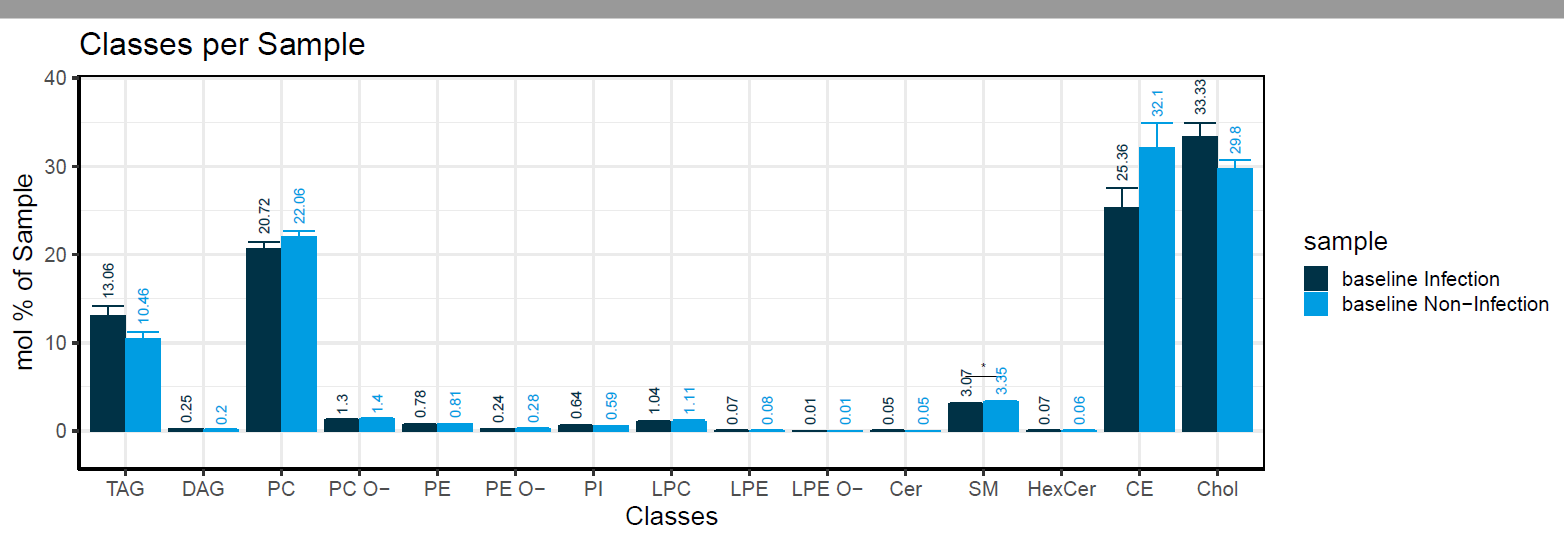


**Supplementary Figure 3.** PCA results of clinical and lipid variables. A: Scree plot. Percentage of variation explained by the top 10 principal components (PCs). B: PCA results of clinical and lipid variables. C: PCA loadings showing the quality of variable representation.

**Supplementary Figure 4.** Area under curve (AUC) results for the prediction of nosocomial infection status. Model 1: Bilirubin D1 levels + Baseline Creatine. Model 2: Model 1 + CE.16.0.0 +CE.18.1.0 +CE.18.2.0. Model 3: Model 2 + Cholesterol. Training dataset 16 infections, 18 non infections. Testing 10 infections, 12 non infections.


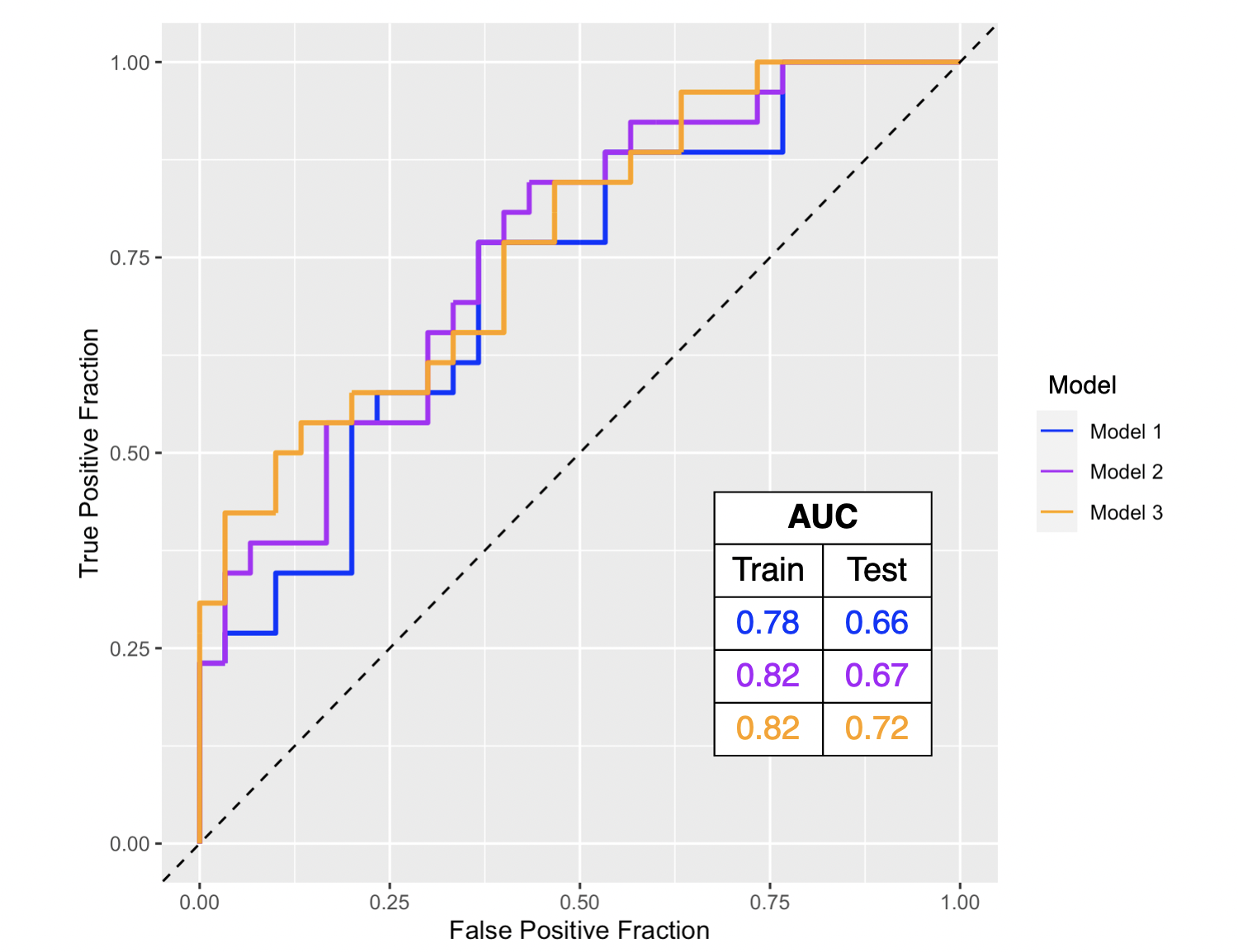


**Supplementary Figure 5.** Correlations between bilirubin and identified lipids identified as important in PLSDA analysis (VIP ≥1) and MELD score. R values show Pearson’s correlation coefficients.


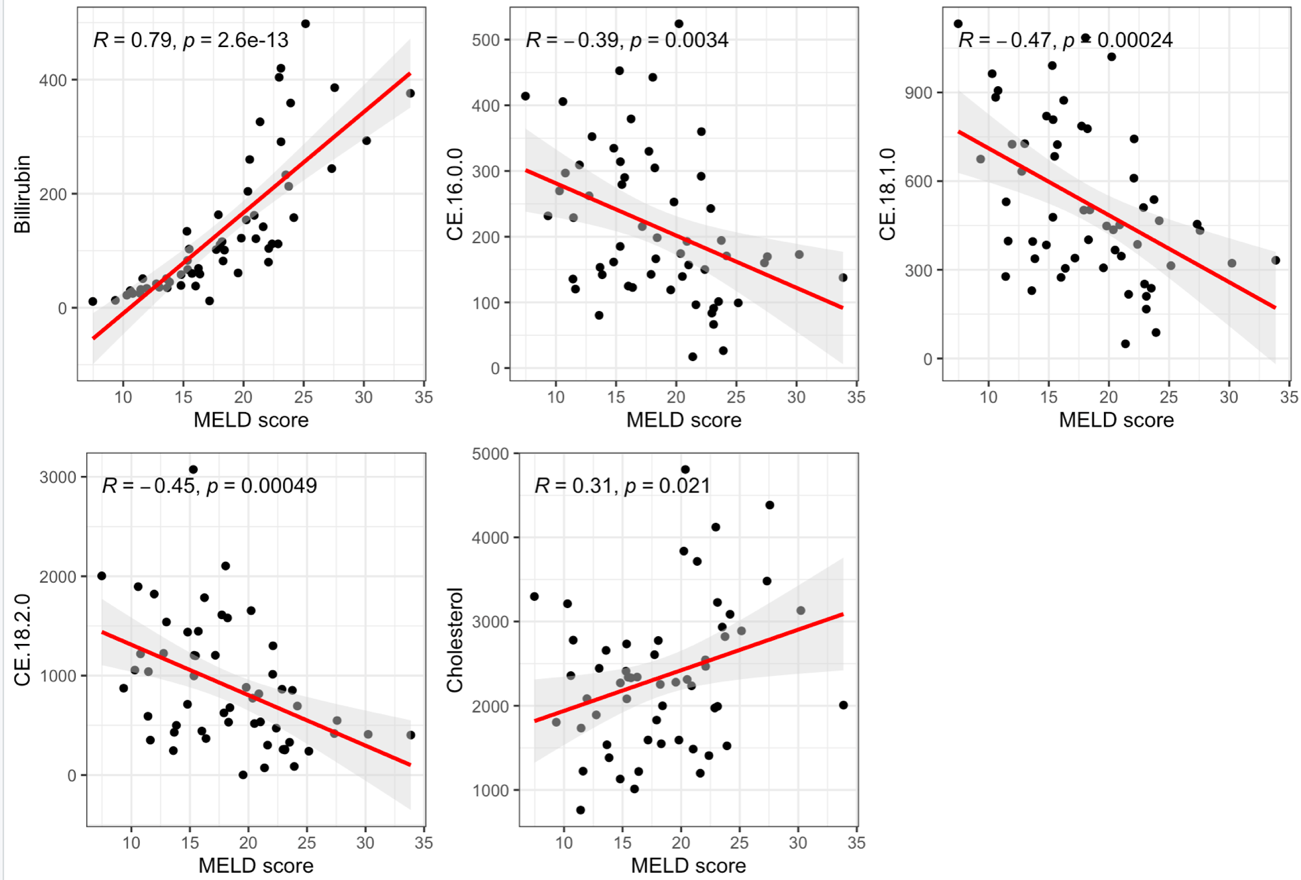


**Supplementary Figure 6.** RNA seq analyses of whole blood untreated and LPS-treated from healthy volunteers (HV, n=5), outpatients with refractory ascites (ORA, n=5) and hospitalised patients with acute decompensation (AD, n=10) for genes involved in lipid metabolomic pathway regulation. Two-way ANOVA with Šídák's multiple comparisons test, *P<0.05, **P<0.01, ***P<0.001.


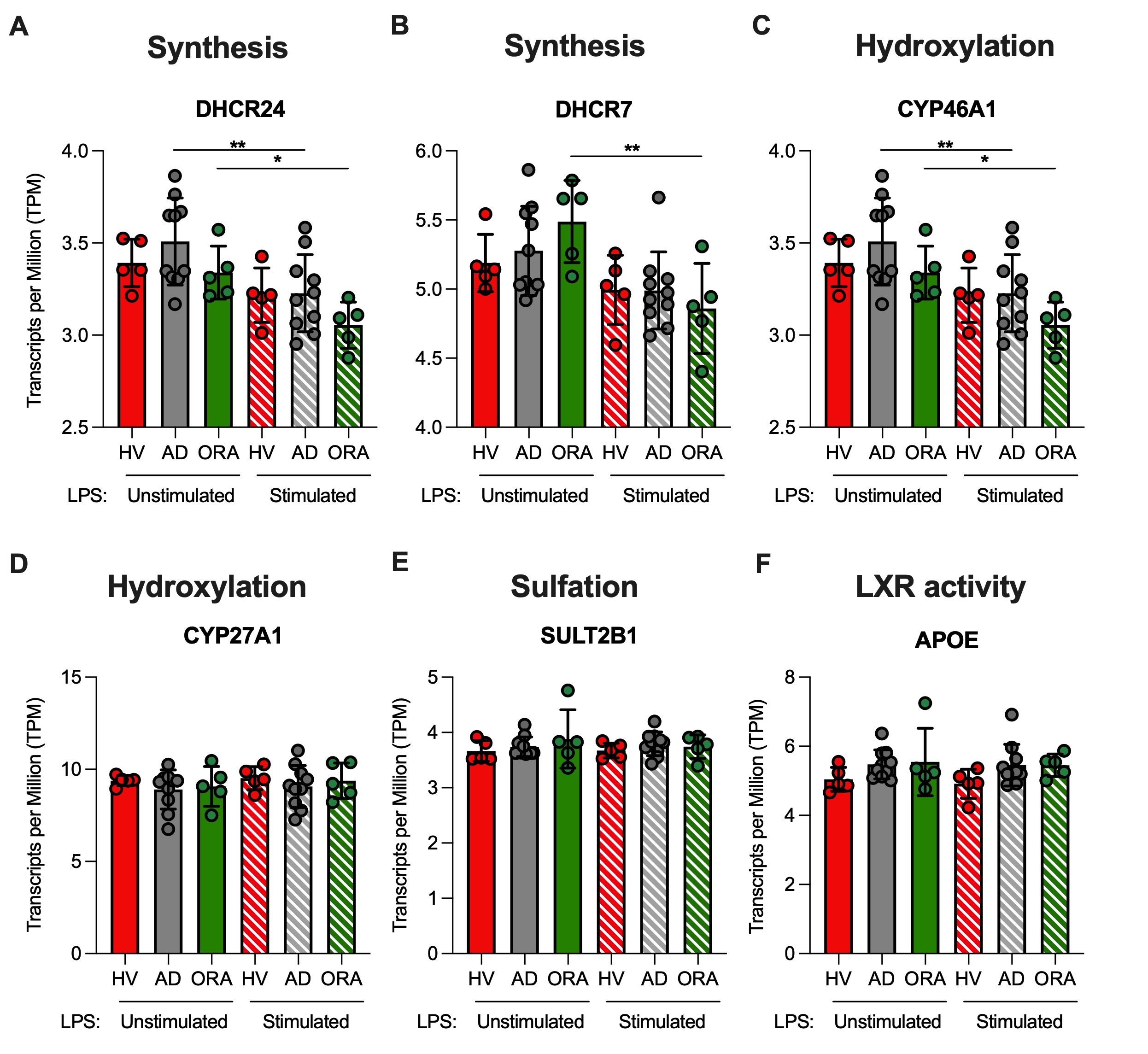


**Supplementary Figure 7.** RNA seq analyses of whole blood untreated and LPS-treated from healthy volunteers (HV, n=5), outpatients with refractory ascites (ORA, n=5) and hospitalised patients with acute decompensation (AD, n=10) for genes involved in inflammation regulation. Two-way ANOVA with Šídák's multiple comparisons test, *P<0.05, **P<0.01, ***P<0.001, ****P<0.0001

**Supplementary Table 1.** Measured analytes with luminex and range of detection are listed in below.

| Measured Analytes | *Detection Range (pg/mL)* |
| --- | --- |
| IL-6 | 1,460 - 2.0 |
| IL-8 | 1,440 - 2.0 |
| TNFα | 4,100 - 5.6 |
| LPS binding protein | 32,990,000 - 45,254 |
| Pro calcitonin | 4,160 - 5.7 |
| Soluble CD14 | 11,344,000 - 15,561 |
| LPS binding protein | 32,990,000 - 45,254 |

**Supplementary Table 2.** Definitions of Extrahepatic Organ Dysfunction

| **Definition of baseline organ dysfunction** | |
| --- | --- |
| Renal | Serum creatinine > 1.5 md/dL |
| Cerebral | Grade III (Drowsy) or grade IV encephalopathy (coma) using the Westhaven Criteria to grade HE |
| Circulatory | Mean Arterial Pressure (MAP)* <60 mmHg or if the patient is receiving inotropic/vasopressor support |
| Respiratory | Sp0_2_/FiO_2_ of <357 |
| **Definition of new organ dysfunction** (Treatment D3 to end of treatment D15) | |
| Renal | Serum creatinine increase ≥50 % compared to value at randomisation, rise in serum creatinine ≥0.3 mg/dL within 48 hours or patient initiated on renal replacement therapy  *Note: patients receiving renal replacement at baseline could not reach this outcome* |
| Cerebral | Grade III (Drowsy) or grade IV encephalopathy (coma) using the Westhaven Criteria to grade HE  *Note: if the patient has grade III HE at baseline they will need to progress to grade IV to reach this endpoint* |
| Circulatory | i) MAP fall to <60 mmHg OR  ii) patient is started on inotropic/vasopressor to support blood pressure  *Note: if MAP <60 mmHg at baseline the inotropic/vasopressor support for blood pressure will need to be initiated to reach endpoint* |
| Respiratory | Any single point increase in Sp0_2_/Fi0_2_ as classified in the following scoring system as compared to Sp0_2_/Fi0_2_ at baseline:   \|  \| 0 \| 1 \| 2 \| \| --- \| --- \| --- \| --- \| \| Sp0_2_/Fi0_2_ \| >357 \| >214 to ≤357 \| ≤214 or mechanical ventilation for respiratory failure \| |

**Supplementary Table 3.** Nosocomial Infections diagnosed in ATTIRE trial patients with no infection and not prescribed antibiotics at hospitalisation that pathogens were reported for.

| Site of infection | Pathogen | Gram Stain | Antimicrobial resistance |
| --- | --- | --- | --- |
| Urinary Tract Infection (UTI) | Coliform | Negative | No |
| UTI | Coliform | Negative | No |
| UTI | E.coli | Negative | No |
| UTI | Coliform | Negative | No |
| UTI | Coliform | Negative | Yes |
| UTI | E.coli | Negative | No |
| UTI | Klebsiella | Negative | No |
| Spontaneous bacteraemia | E.coli | Negative | No |
| Spontaneous bacteraemia | Acinetobacter | Negative | No |
| Soft tissue/skin infection | Staph aureus | Positive | No |
| Soft tissue/skin infection | Staph aureus | Positive | Not reported |
| Spontaneous Bacterial Peritonitis (SBP) | Gram positive cocci | Positive | Not reported |
| SBP | Strep Gallolyticus ssp. pasteurianus | Positive | No |
| SBP | Enterococcus faecium, Citrobacter farmeri, Veillonella atypica | Positive | Yes |
| SBP | Gram +ve cocci | Positive | No |
| Spontaneous bacteraemia | Staph aureus | Positive | No |
| Spontaneous bacteraemia | E.faecalis | Positive | No |
| Spontaneous bacteraemia | Staph aureus | Positive | No |
| Spontaneous bacteraemia | Streptococcus | Positive | No |
| Lower respiratory tract infection | Staphylococcus  (uncharacterised) | Positive | Not reported |
| Lower respiratory tract infection | Influenza A | N/A | N/A |

**Supplementary Table 4.** Multivariate models to investigate ability of clinical characteristics of the 360 patients without an infection and not prescribed antibiotics at ATTIRE trial baseline to predict subsequent nosocomial infection. SE-standard error, OR-odds ratio.

| **Variable** | **β** | **SE** | **OR** | **95% Lower** | **95% Upper** | ***P* value** |
| --- | --- | --- | --- | --- | --- | --- |
| **(Intercept)** | **-1.794** | **1.281** | **0.166** | **0.013** | **2.003** | **0.161** |
| **Bilirubin (ummol/L)** | 0.003 | 0.001 | 1.003 | 1.001 | 1.005 | **0.006** |
| **Age (years)** | 0.014 | 0.015 | 1.014 | 0.985 | 1.045 | 0.353 |
| **Gender** | -0.065 | 0.319 | 0.937 | 0.493 | 1.732 | 0.840 |
| **Albumin (g/L)** | -0.034 | 0.039 | 0.966 | 0.895 | 1.045 | 0.381 |
| **Creatinine (mmol/L)** | -0.002 | 0.004 | 0.998 | 0.991 | 1.005 | 0.678 |
| **WCC (x109/L)** | 0.017 | 0.032 | 1.017 | 0.952 | 1.081 | 0.589 |
| **CRP (mg/L)** | 0.001 | 0.007 | 1.001 | 0.987 | 1.015 | 0.860 |
|  |  |  |  |  |  |  |
| **(Intercept)** | -1.988 | 1.325 | 0.137 | 0.010 | 1.793 | 0.134 |
| **Bilirubin (ummol/L)** | 0.003 | 0.001 | 1.003 | 1.001 | 1.005 | 0.010 |
| **Age (years)** | 0.013 | 0.015 | 1.013 | 0.983 | 1.045 | 0.388 |
| **Gender** | -0.004 | 0.322 | 0.996 | 0.521 | 1.853 | 0.990 |
| **Albumin (g/L)** | -0.023 | 0.041 | 0.978 | 0.903 | 1.060 | 0.580 |
| **Creatinine (mmol/L)** | -0.001 | 0.004 | 0.999 | 0.991 | 1.006 | 0.766 |
| **WCC (x109/L)** | 0.025 | 0.033 | 1.025 | 0.958 | 1.091 | 0.452 |
| **CRP (mg/L)** | 0.001 | 0.007 | 1.001 | 0.986 | 1.015 | 0.883 |
| **VB** | 0.020 | 0.605 | 1.020 | 0.272 | 3.085 | 0.973 |
| **Presence of Ascites** | -0.290 | 0.311 | 0.748 | 0.408 | 1.388 | 0.351 |
| **HE** | -0.316 | 0.466 | 0.729 | 0.272 | 1.734 | 0.498 |

**Supplementary Table 5.** Multivariate model to investigate ability of a serum bilirubin threshold >188 in the 360 patients without an infection and not prescribed antibiotics at ATTIRE trial baseline to predict subsequent nosocomial infection. SE-standard error, OR-odds ratio.

| **Variable** | **β** | **SE** | **OR** | **95% Lower** | **95% Upper** | ***P* value** |
| --- | --- | --- | --- | --- | --- | --- |
| **Bilirubin**  **threshold** | 1.025 | 0.352 | 2.786 | 1.396 | 5.575 | **0.004** |
| **Age (years)** | 0.014 | 0.016 | 1.014 | 0.983 | 1.046 | 0.384 |
| **Gender** | -0.058 | 0.327 | 0.944 | 0.489 | 1.770 | 0.860 |
| **Albumin (g/L)** | -0.021 | 0.041 | 0.980 | 0.905 | 1.063 | 0.614 |
| **Creatinine (mmol/L)** | -0.001 | 0.004 | 0.999 | 0.991 | 1.006 | 0.722 |
| **WCC (x109/L)** | 0.026 | 0.034 | 1.026 | 0.958 | 1.094 | 0.438 |
| **CRP (mg/L)** | 0.000 | 0.007 | 1.000 | 0.985 | 1.014 | 0.984 |
| **VB** | -0.045 | 0.618 | 0.956 | 0.249 | 2.974 | 0.942 |
| **Presence of Ascites** | -0.229 | 0.316 | 0.795 | 0.430 | 1.491 | 0.469 |
| **HE** | -0.331 | 0.470 | 0.718 | 0.266 | 1.722 | 0.481 |
| **NSBB use** | 0.094 | 0.394 | 1.099 | 0.491 | 2.326 | 0.811 |
| **PPI use** | 0.228 | 0.307 | 1.257 | 0.693 | 2.317 | 0.457 |
| **Prednisolone use** | 0.103 | 0.400 | 1.109 | 0.496 | 2.397 | 0.796 |

**Supplementary Table 6.** Baseline clinical characteristics of patients with plasma samples analysed for biomarkers of bacterial translocation, infection and inflammation. Data shown as mean (95% CI) for age with unpaired t-test as normally distributed but median (95% CI) for other clinical variables with Mann-Whitney t-test, as data not normally distributed. Differences between gender, use of PPI/NSBB/prednisolone and alcoholic hepatitis diagnosis assessed using chi-squared testing.

|  | Developed Nosocomial Infection (n=16) | Did not develop Nosocomial Infection (n=52) | *P* value |
| --- | --- | --- | --- |
| Male | 13 (81.3%) | 38 (73.1%) | 0.51 |
| Alcohol-induced cirrhosis | 14 (87.5%) | 41 (79%) | 0.44 |
| MELD | 19.8 (15.7-22.1) | 20.9 (19-23.1) | 0.4 |
| Bilirubin (umol/L) | 116 (60-244) | 129.5 (96-174) | 0.77 |
| Creatinine (mmol/L) | 66 (59-71) | 73.5 (64-86) | 0.18 |
| Albumin (g/L) | 23 (22-24) | 24 (22-25) | 0.2 |
| WCC (x109/L) | 6.8 (5.3-7.7) | 6.85 (6.2-8.4) | 0.54 |
| CRP (mg/L) | 12 (5-31) | 22 (14-36) | 0.08 |
| Age (years) | 53.3 (48.5-58) | 54.6 (51.1-58) | 0.68 |
| Albumin treatment group | 8 (50%) | 23 (44%) | 0.69 |
| PPI use | 7 (44%) | 29 (55.8%) | 0.4 |
| NSBB use | 2 (12.5%) | 10 (19.2%) | 0.54 |
| Prednisolone use | 4 (25%) | 13 (25%) | 1 |
| Alcoholic hepatitis | 5 (31.3) | 16 (30.8%) | 0.97 |
| Hepatic Encephalopathy (Grade III/IV) | 1 (6.25%) | 8 (15.4%) | 0.35 |
| Variceal Bleed | 1 (6.25%) | 3 (5.8%) | 0.94 |

**Supplementary Table 7.** Baseline clinical characteristics of 56 patient subcohort of those with no infection at baseline and not treated with antibiotics with plasma samples analysed for lipid metabolomic profiling. Data shown as mean (95% CI) for age with unpaired t-test as normally distributed but median (95% CI) for other clinical variables with Mann-Whitney t-test, as data not normally distributed. Differences between gender, use of PPI/NSBB/prednisolone and alcoholic hepatitis diagnosis assessed using chi-squared testing.

|  | Developed Nosocomial Infection (n=26) | Did not develop Nosocomial Infection (n=30) | *P* value |
| --- | --- | --- | --- |
| Male | 15 (57.7%) | 16 (53.3%) | 0.74 |
| Alcohol-induced cirrhosis | 21 (80.7%) | 16 (80%) | 0.94 |
| MELD | 19.05 (15.7-23.1) | 16.77 (13-20.9) | 0.10 |
| Bilirubin (umol/L) | 128 (82-260) | 68 (42-112) | **0.03** |
| Creatinine (mmol/L) | 67.5 (57-77) | 68.5 (55-75) | 0.87 |
| Albumin (g/L) | 23 (22-24) | 23 (20-24) | 0.70 |
| WCC (x109/L) | 6.85 (5.3-8.1) | 7.2 (6.1-8.4) | 0.60 |
| CRP (mg/L) | 14 (6-20) | 18 (9-21) | 0.40 |
| Age (years) | 52.6 (46.1-58.1) | 57.2 (52.2-62) | 0.11 |
| Albumin treatment group | 10 (38.5%) | 15 (50%) | 0.38 |
| PPI use | 8 (30.8%) | 10 (33.3%) | 0.84 |
| NSBB use | 5 (19.2%) | 5 (16.7%) | 0.80 |
| Prednisolone use | 7 (26.9%) | 3 (10%) | 0.10 |
| Alcoholic hepatitis | 9 (34.6%) | 5 (16.7%) | 0.12 |
| Hepatic Encephalopathy  (Grade III/IV) | 2 (7.7%) | 3 (10%) | 0.76 |
| Variceal bleed | 2 (7.7%) | 2 (6.7%) | 0.88 |

**Supplementary Table 8.** Details of participant cohorts for RNA seq analyses – healthy volunteers (HV), day case paracentesis patients and hospitalised with acute decompensation of cirrhosis (median and IQR values).

|  | Healthy volunteers | Day Case Paracentesis patients | Hospitalised with acute decompensation of cirrhosis |
| --- | --- | --- | --- |
| Number | 5 | 5 | 10 |
| Cause of cirrhosis | N/A | Alcohol 4  NASH 1 | Alcohol 8  NASH 2 |
| MELD | N/A | 13 (10-15) | 17 (12-23) |
| Bilirubin | 10 (7-12) | 26 (16-29) | 60 (35-86) |
| Creatinine | 75 (66-96) | 107 (58-138) | 90 (56-100) |
| Albumin | 49 (46-50) | 33 (32-35) | 28 (26-32) |
| WCC | 5.7 (4.1-6.8) | 4.5 (3-5.4) | 8.6 (6.4-13.8) |
| CRP | 1.1 (0-1.25) | 8 (3-13) | 24.2 (15.78-42.93) |
| Age (years) | 28.5 (24.5-39) | 60 (55.25-66.25) | 56.5 (53.25-67.5) |
| Male | 80% | 80% | 70% |
